# Culture-Independent Detection and Genotyping of *Mycoplasma pneumoniae* in Clinical Specimens from Beijing, China

**DOI:** 10.1371/journal.pone.0141702

**Published:** 2015-10-28

**Authors:** Fei Zhao, Liyong Liu, Xiaoxia Tao, Lihua He, Fanliang Meng, Jianzhong Zhang

**Affiliations:** 1 State Key Laboratory of Infectious Disease Prevention and Control, National Institute for Communicable Disease Control and Prevention, Chinese Center for Disease Control and Prevention, Beijing, China; 2 Collaborative Innovation Center for Diagnosis and Treatment of Infectious Diseases, Hangzhou, Zhejiang, China; Iowa State University, UNITED STATES

## Abstract

A duplex real-time PCR assay was designed for simultaneous detection and genotyping of *Mycoplasma pneumoniae* (*M*. *pneumoniae*). The detection/typing performance of this duplex PCR method, targeting specific genes for *M*. *pneumoniae* type 1 (*mpn 459*) and type 2 (*mpna 5864*), was compared to that of the previously published MpP1 real-time PCR assay and the genotyping method for the adhesin P1 gene (*mpn 141*). A total of 1,344 throat swab specimens collected from patients in Beijing, China were tested for *M*. *pneumoniae* by bacterial culture, MpP1 real-time PCR assay, and our duplex PCR assay, and positive detection rates of 26.9%, 34.4%, and 33.7%, respectively, were obtained. The duplex PCR method demonstrated high sensitivity and accuracy for detecting and genotyping *M*. *pneumoniae*, and significant differences in genotyping ability were observed when compared to the conventional P1 gene-based method. *M*. *pneumoniae* type 1 was the predominate genotype from 2008 to 2012 in Beijing, and a shift from type 1 to type 2 began to occur in 2013. To our knowledge, this is the first reported incidence of a type shift phenomenon of *M*. *pneumoniae* clinical isolates in China. These genotyping results provide important information for understanding recent changes in epidemiological characteristics of *M*. *pneumoniae* in Beijing.

## Introduction

*Mycoplasma pneumoniae* (*M*. *pneumoniae*), a small prokaryote devoid of a cell wall, is one of the most common etiological agents of human respiratory tract infections. About 10–40% of all cases of community acquired pneumonia can be attributed to *M*. *pneumoniae*, which occurs in epidemic peaks at intervals of 3–7 years [1–3]. Occasionally, severe cases with extrapulmonary involvement can result in hospitalization and death due to neurological disease, such as encephalitis [4, 5]. The high rate of morbidity and the occasional mortality reinforces the need for timely diagnosis. While the culturing of *M*. *pneumoniae* is time-consuming (2–8 weeks) and unreliable, serological detections require the availability of paired sera exhibiting IgG antibodies, which is age- and time-dependent [6]. Molecular approaches, especially real-time PCR methods, are currently used for rapid, sensitive, and specific detection of *M*. *pneumoniae* in clinical specimens [7–10].

In addition to detection, typing of clinical isolates is practically important for understanding the epidemiology of *M*. *pneumoniae* infections and for analysis of endemic outbreaks [11, 12]. In general, *M*. *pneumoniae* is regarded as a genetically highly uniform species. Previous investigations have revealed 2 types of clinical isolates (type 1 and type 2) that significantly differ in their sequences of the P1gene, *mpn 141* [13]. This P1 gene, which codes for an important 170-kDa adhesin and antigenic factor, is the most frequently used typing target for *M*. *pneumoniae*. Typing schemes based on conventional PCR [14], restriction fragment length polymorphism [15], real-time PCR with high-resolution melt-analysis [16], and sequencing [17] have been developed to differentiate the P1 genotypes of *M*. *pneumoniae*. About 8% of the *M*. *pneumoniae* genome consists of multiple copies of four RepMP elements [18–20], two of which (RepMP 2/3 and RepMP 4) are within *mpn 141*. Besides the two major *M*. *pneumoniae* types, in-depth analysis of the RepMp2/3 and RepMp4 repeat sequences within the P1 gene has revealed one sequence variant of type 1 (i.e., variant 1 or V1) [21] and four sequence variants of type 2 (i.e., V2a, b, c, and d) [17, 22–24]. This variation can be generated by homologous recombination of the RepMP elements located both inside and outside the P1 gene [21, 25]. Preservation of these repetitive sequences during presumed genome minimalization reinforces their importance, as they form pools of sequences for homologous recombinations that yield antigenic variations of *M*. *pneumoniae* proteins. It is therefore highly likely that novel variants of the P1 gene will continue to appear with homologous recombination, thereby increasing the ambiguity of the traditional P1 typing approach. Furthermore, some studies have indicated that typing targets other than the P1 gene may exist. Musatovova et al. [20] revealed that the sequence divergence involving the RepMP 1-containing genes (*mpn 130*, *mpn 137*, and *mpn 138*), is strictly two types specific. Spuesens et al. [26] and Catrein et al. [27] also confirmed two highly conserved groups of *M*. *pneumoniae* strains based on sequence divergence of the RepMP5-containing gene, *mpn 142*.

In recent years, attempts have been made to find additional proteomic or genomic markers for typing of *M*. *pneumoniae*. Pereyre et al. [28] and our group [29] classified *M*. *pneumoniae* strains into two distinct groups, based on polypeptide levels, by matrix-assisted laser desorption ionization–time of flight mass spectrometry (MALDI-TOF MS). Genomic analyses [30, 31] have also demonstrated that *M*. *pneumoniae* strains can be classified into two distinct types and further classified into sequence variants of each type. This notion was supported by the analysis of phylogenetic trees generated from all RepMP2/3 and RepMP4 sequences. In each of these trees, clear separation was observed between the type 1 and type 2 strains [25]. In 2015, two reports of the whole-genome analysis of 38 *M*. *pneumoniae* were published [32, 33]. Whole-genome sequences were obtained by sequencing 20 clinically isolated strains, including type 1, type 2, V2a, and V2c strains, at our laboratory (unpublished data). We compared our genome sequences with the following *M*. *pneumoniae* whole-genome sequences reported on the National Center for Biotechnology Information (NCBI) website: M129 (GenBank: U00089.2), FH (GenBank: CP002077.1), resequenced FH (GenBank: CP010546.1), 309 (GenBank: AP012303.1), M29 (GenBank: CP008895.1), M129-B7 (GenBank: CP003913.2), PO1 (GenBank: CP010551.1), 19294 (GenBank: CP010539.1), 39443 (GenBank: CP010540.1), 85138 (GenBank: CP010545.1), 85084 (GenBank: CP010544.1), 54524 (CP010543.1), 54089 (CP010542.1), 51494 (CP010541.1), MAC (CP010550.1), M2592 (GenBank: CP010549.1), M2192 (GenBank: CP010548.1), M1139 (GenBank: CP010547.1), and PI1428 (GenBank: CP010538.1). We found that the genome sequences exhibited distinct characteristics consistent with two biological types of *M*. *pneumoniae*. Taken together, these previous reports confirm the existence of two highly conserved types of *M*. *pneumoniae* strains, each with respective variants based on minor sequence variations in the P1 gene.

Nevertheless, broad application of the aforementioned conventional bacterial typing schemes is hampered by the time-consuming and unreliable cultivation of *M*. *pneumoniae* from clinical specimens. Thus, these lengthy typing methods have low sensitivity compared to real-time PCR performed directly in clinical specimens without cultivation of *M*. *pneumoniae*. To simplify the detection/typing procedure and increase sensitivity, our aims were to develop a culture-independent, duplex real-time PCR method for detecting and typing *M*. *pneumoniae* simultaneously in clinical specimens and to evaluate the detection/typing performance of this method by comparison to that of conventional methods.

## Materials and Methods

### Ethics statement

The study was approved by the Research Ethics Committee of the National Institute for Communicable Disease Control and Prevention (ICDC). No specimens were collected for this study. We used 1344 preserved throat swab specimens that were obtained in our laboratory during previous studies. The consent procedure for obtaining the throat samples was approved by the ethics board committee of Beijing Chao-Yang Hospital, Beijing Children’s Hospital, and Beijing Centers for Disease Control and Prevention, separately. Because throat swab is noninvasive, verbal consent was obtained from all patients or their guardians (for minor patients).

### Clinical specimens and strains

From August 2008 to December 2014, a total 1344 throat swab specimens from 388 pediatric (< 14 years of age) and 956 adolescent and adult patients were collected from Beijing Chao-Yang Hospital, Beijing Children’s Hospital, and Beijing Centers for Disease Control and Prevention. All clinical specimens were obtained from respiratory tract infection patients according to clinical symptoms. The following American Type Culture Collection (ATCC) reference and clinically isolated strains were used to assess the sensitivity, specificity, lowest detectable limit (LDL), and typing ability of the duplex real-time PCR method: *M*. *pneumoniae* (ATCC 29342, 15531, 39505, 29343, 29085, 15377, 15492, 15293, and 49894; ATCC, Manassas, VA), *Mycoplasma salivarium* (ATCC 23064), *Mycoplasma orale* (ATCC 23714), *Mycoplasma faucium* (ATCC 25293), *Mycoplasma genitalium* (ATCC 33530), *Ureaplasma urealyticum* (ATCC 27618), *Mycoplasma fermentans* (ATCC 19989), *Mycoplasma hominis* (ATCC 23114), *Mycoplasma penetrans* (ATCC 55252), *Mycoplasma hyorhinis* (ATCC 17981), *Mycoplasma pirum* (ATCC 25960), *Escherichia coli* (ATCC 11229), *Streptococcus pneumoniae* (ATCC 49619), *Staphylococcus aureus* (ATCC 29213), *Staphylococcus epidermidis* (ATCC 35984), *Pseudomonas aeruginosa* (clinical isolate), *Chlamydophila pneumoniae* (clinical isolate), *Mycobacterium tuberculosis* (clinical isolate), *Legionella pneumophila* (clinical isolate), *Haemophilus influenzae* (clinical isolate), *Neisseria meningitidis* (clinical isolate), and 56 other *M*. *pneumoniae* clinical isolates.

### Duplex real-time PCR assay

By comparing the genome sequences of 20 *M*. *pneumoniae* clinical isolates with those of the reference strains, we observed remarkable differences in large fragment insertions or deletions, mainly between genes *mpn* 459 (corresponding to the type 1 strain, M129) and *mpna* 5864 (corresponding to the type 2 strain, 309). The type 1 strains had an approximately 3-kb insertion encoding three genes, *mpn* 457–459, whereas the type 2 strains had an approximately 5-kb insertion encoding five genes, *mpna* 5861–5865. Therefore, the primers and probes, designed with Primer Express software (version 3.0; Life Technologies-Applied Biosystems, Grand Island, NY, USA), were based on the conserved sequences of the 3kb and 5kb insertion fragments within the type 1 and type 2 strain, respectively. These primers and probes are listed in Table 1. Each PCR mixture was prepared in a total volume of 25 μl and contained the following per reaction: 12.5 μl Platinum Quantitative PCR SuperMix-UDG (Life Technologies-Invitrogen), 1.5 μl MgCl_2_ (50 mM), 0.5 μM final concentration of each primer, 0.2 μM final concentration of each probe, 1.25 U Platinum Taq DNA polymerase (5 U/μl; Life Technologies-Invitrogen), 1 μl PCR nucleotide mix (10 mM), 5 μl nucleic acid extracted from each specimen, and nuclease-free water to achieve a 25 μl final volume. Real-time PCR for each target was performed in the CFX96 Real-time PCR Detection System (Bio-Rad, Hercules, CA, USA) under the following conditions: predenaturation at 95°C for 2 min, followed by 45 cycles at 95°C for 15 s and 56°C for 15 s. The data were analyzed with the CFX Manager Software (version 2.1; Bio-Rad).

**Table 1:** Primers and probes used in the duplex real-time PCR assay.

Primer/probe name	Sequence (5’→3’)	Target gene
Mptype1-F	CCAGATTCACGTTTAATTTC	*mpn*459^a^
Mptype1-R	GCATCTAACATGAAGACTG	*mpn*459
Mptype1-P	FAM-AACCAACAACTTCTCATTCATCCTCAG-BHQ1	*mpn*459
Mptype2-F	TTGGGTAAACCTAATTTGC	*mpna*5864^b^
Mptype2-R	ACACGTATTAGCATCACTA	*mpna*5864
Mptype2-P	VIC-AAGACTATTCGCCTTACAACCAACC-BHQ1	*mpna*5864

^a^Found in *M*. *pneumoniae* type 1 strain, M129.

^b^Found in *M*. *pneumoniae* type 2 strain, 309.

### LDL, sensitivity, and specificity of duplex real-time PCR assay

DNA was extracted from the reference strains and clinical isolates mentioned above with the QIAamp DNA Blood Mini Kit (QIAGEN, Hilden, Germany). The standard curves and LDL for each *M*. *pneumoniae* type were determined by analyzing serial 10-fold dilutions of Tris-buffered DNA extracted from *M*. *pneumoniae* type 1 (M129 strain [ATCC29342], 0.162 fg/μl to 0.162 ng/μl) and *M*. *pneumoniae* type 2 (FH strain [ATCC 15531] 0.183 fg/μl to 0.183 ng/μl). *M*. *pneumoniae* type 1 DNA was detected with FAM fluorescence, and type 2 DNA was detected with VIC fluorescence. Each dilution of DNA was assayed in triplicate.

### DNA mixtures for duplex real-time PCR detection analysis

The detection ability was determined by analyzing the following four mixtures comprised of different compositions of ATCC 29342 and ATCC 15531 DNA: mixture A (1.62 pg/μl ATCC 29342 and 1.83 fg/μl ATCC 15531), mixture B (1.62 fg/μl ATCC 29342 and 1.83 pg/μl ATCC 15531), mixture C (1.62 pg/μl ATCC 29342 and 1.83 pg/μl ATCC 15531), and mixture D (1.62 fg/μl ATCC 29342 and 1.83 fg/μl ATCC 15531). Each DNA mixture was assayed in triplicate.

### *M*. *pneumoniae* strains for comparative typing analysis by culture-based and duplex real-time PCR methods

DNA extracted from 9 *M*. *pneumoniae* ATCC strains and 56 clinical isolates were amplified with the duplex real-time PCR method for typing analysis. All 65 *M*. *pneumoniae* strains were genotyped by full-length sequencing of the P1 gene [23] with the amplification primers SeqP1-F (5’-ATGCACCAAACCAAAAAAACTGCCT-3’) and SeqP1-R (5’-CTAAGCGGGTTTTTTAGGTGGTTGC-3’). The same *M*. *pneumoniae* strains were also genotyped by MALDI-TOF MS, based on their peptide mass fingerprints, as described in our previous report [29].

### Analysis of *M*. *pneumoniae* detection and typing in clinical specimens

A total of 1344 throat swab specimens were cultured with selective Mycoplasma Broth (Thermo Scientific-Oxoid Limited, Basingstoke Hampshire, UK). Each culture-positive specimen was subcultured and purified by the filtration-cloning technique for *M*. *pneumoniae* clinical isolates. Genomic DNA of each isolate was then extracted with the QIAamp DNA Blood Mini Kit, and subsequently genotyped with the P1 gene-based PCR method as previously reported [12, 32]. The duplex real-time PCR assay and the previously reported MpP1 real-time PCR assay [34] were used simultaneously to detect *M*. *pneumoniae* DNA in the throat swab specimen extracts.

### Statistical analysis

Quantitative data are presented as the mean ± standard deviation (SD). The results were calculated in the Statistical Analysis System software package (version 9.3; Cary, NC, USA). Differences in number of positive results between each two of the three detection/typing methods were tested with the paired chi-square test. Differences in *M*. *pneumoniae* typing differentiation ability and in number of positive typing results between the P1 gene-based PCR method and the duplex PCR method were tested with the chi-square test and paired chi-square test, respectively, with a *P* value < 0.01 considered statistically significant.

## Results

### LDL, sensitivity, and specificity of duplex real-time PCR assay

Based on the crossing threshold (CT) values for the serial 10-fold dilutions of DNA, the LDLs of *M*. *pneumoniae* type 1 and type 2 were approximately 8.1 fg and 9.3 fg DNA, respectively (Table 2). Both these amounts are equivalent to approximately 3 colony forming units (CFU). The standard curves (CT vs. log CFU) for *M*. *pneumoniae* type 1 and type 2, generated by the serial 10-fold dilutions, yielded coefficient of determination (r^2^) values of 0.996 and 0.997, respectively. The duplex real-time PCR assay amplified DNA from all *M*. *pneumoniae* species tested (9 ATCC strains and 56 clinical isolates). This assay did not amplify DNA from any of the other Mycoplasma species, other respiratory bacteria (10 mollicute species and 10 common respiratory bacteria, listed in Materials and Methods), or human DNA, even when tested at concentrations (at ng level) markedly higher than the LDL.

**Table 2:** CT values of serial 10-fold dilutions of *M*. *pneumoniae* DNA analyzed by duplex real-time PCR.

		CT value^a^	
*M*. *pneumoniae* strain	CFU (DNA quantity)	Type 1 (FAM fluorescence)	Type 2 (VIC fluorescence)
ATCC 29342	3×10^5^ CFU (0.8 ng DNA)	17.65±0.10	N^b^
	3×10^4^ CFU (80 pg DNA)	21.04±0.05	N
	3×10^3^CFU (8 pg DNA)	24.58±0.16	N
	3×10^2^ CFU (800 fg DNA)	29.29±0.04	N
	30 CFU (80 fg DNA)	33.50±0.12	N
	3 CFU (8 fg DNA)	38.17±0.31	N
	0.3 CFU (0.8 fg DNA)	N	N
ATCC 15531	3×10^5^ CFU (0.9 ng DNA)	N	15.34 ± 0.08
	3×10^4^ CFU (90 pg DNA)	N	18.75 ± 0.04
	3×10^3^ CFU (9 pg DNA)	N	22.83 ± 0.14
	3×10^2^ CFU (900 fg DNA)	N	27.39 ± 0.11
	30 CFU (90 fg DNA)	N	31.95 ± 0.36
	3 CFU (9 fg DNA)	N	35.93 ± 0.66
	0.3 CFU (0.9 fg DNA)	N	N

^a^CT values expressed as the mean ± SD of 3 replicates per dilution.

^b^N, negative result.

### Duplex real-time PCR detection analysis with DNA mixtures

Mixture C, with higher (pg) concentrations of *M*. *pneumoniae* type 1 and type 2 DNA, detected positive for both types, but the CT for each fluorescent (FAM and VIC) signal was delayed about 1–2 cycles compared to that for each signal of amplified *M*. *pneumoniae* DNA detected individually at the same concentration. Mixture D, with lower (fg) concentrations of type 1 and type 2 DNA, detected positive for both types only once. In mixtures A and B, with different concentrations of type 1 and type 2 DNA, only the type with the higher concentration could be detected (Table 3).

**Table 3:** CT values of 4 mixtures of *M*. *pneumoniae* type 1 and type 2 DNA analyzed by duplex real-time PCR.

	CT values by mixed DNA analysis		CT values by individual DNA analysis	
Composition of *M*. *pneumoniae* DNA mixture ^a^	ATCC 29342 (Type 1)	ATCC 15531 (Type 2)	ATCC 29342 (Type 1)	ATCC 15531 (Type 2)
A	26.09	N ^b^	24.60	36.66
	25.89	N	24.72	35.37
	25.74	N	24.41	35.77
B	N	23.43	38.26	22.69
	N	23.45	37.83	22.97
	N	22.99	38.42	22.82
C	26.04	23.53	24.60	22.69
	25.89	23.37	24.72	22.97
	25.78	23.12	24.41	22.82
D	N	41.12	38.26	36.66
	39.77	38.86	37.83	35.37
	N	N	38.42	35.77

^a^ A: 8 pg ATCC 29342+9 fg ATCC 15531; B: 8 fg ATCC 29342+9 pg ATCC 15531; C: 8pg ATCC 29342+9 pg ATCC 15531; D: 8 fg ATCC 29342+9 fg ATCC 15531.

^b^ N, negative result.

### Comparative *M*. *pneumoniae* typing analysis by culture-based and duplex real-time PCR methods

Duplex real-time PCR typing of 9 *M*. *pneumoniae* ATCC strains and 56 clinical isolates revealed 44 type 1 strains and 21 type 2 strains. These typing results were completely consistent with those produced by MALDI-TOF MS and P1 gene-based typing. The 21 type 2 strains revealed by the P1 gene-based method comprised of three traditional type 2, two V2a, and 16 V2c strains, based on the minor variations in the P1 gene. No variant 1 strain was detected (Table 4).

**Table 4:** Genotypes of 65 *M*. *pneumoniae* strains by culture-based and duplex real-time PCR methods.

*M*. *pneumoniae* strain	P1 gene	MALDI-TOF MS	Duplex real-time PCR
ATCC 49894	2	2	2
ATCC 39505	1	1	1
ATCC 29343	1	1	1
ATCC 29342	1	1	1
ATCC 29085	1	1	1
ATCC 15531	2	2	2
ATCC 15492	2	2	2
ATCC 15377	1	1	1
ATCC 15293	1	1	1
P005	1	1	1
U014	1	1	1
P089	1	1	1
P074	2a	2	2
P054	2c	2	2
P053	2c	2	2
P042	2c	2	2
P037	1	1	1
P036	2c	2	2
P033	2c	2	2
P028	2a	2	2
P169	1	1	1
P164	1	1	1
P160	1	1	1
P015	1	1	1
P146	1	1	1
P118	2c	2	2
F170	2c	2	2
21309	1	1	1
21133	1	1	1
21121	1	1	1
21111	1	1	1
21109	2c	2	2
21101	1	1	1
21077	2c	2	2
21065	1	1	1
21059	2c	2	2
21038	1	1	1
21024	1	1	1
21022	1	1	1
21009	1	1	1
12066	1	1	1
12039	1	1	1
12012	1	1	1
12010	2c	2	2
434	1	1	1
429	1	1	1
429	1	1	1
388	1	1	1
385	1	1	1
373	1	1	1
370	1	1	1
159	2c	2	2
145	1	1	1
137	1	1	1
105	1	1	1
090	1	1	1
089	1	1	1
023	1	1	1
H123	1	1	1
H080	2c	2	2
H088	2c	2	2
H108	1	1	1
H140	1	1	1
H098	2c	2	2
H187	2c	2	2

### *M*. *pneumoniae* detection and typing analysis in clinical specimens

Of the 1344 throat swab specimens, the *M*. *pneumoniae* positive detection rates of the culture method, MpP1 real-time PCR assay, and our duplex real-time PCR assay were 26.9% (362/1344), 34.4% (462/1344), and 33.7% (453/1344), respectively. The positive detection rates of the MpP1 and duplex real-time PCR assays were not significantly different (paired χ^2^, *P* = 0.0389). Differences were observed between those of the culture method and each of the PCR methods (both paired χ^2^, *P* < 0.01). A total of 356 specimens tested positive by all three methods, 92 specimens tested positive by the two real-time PCR methods, and only 4 specimens tested positive by the culture method alone (Table 5). Of the 362 *M*. *pneumoniae* isolates cultured from the throat swab specimens, 98 were genotyped by full-length sequencing of the P1 gene, and the other 264 isolates were genotyped by the P1 gene-based PCR method [12, 35]. Based on genotyping analysis by the culture-based methods, 85.4% (309/362) of the isolates were traditional type 1 (no variant 1 detected), and 14.6% (53/362) were type 2 (2 isolates were variant 2a, and 51 isolates were variant 2c).

**Table 5:** Positive results for *M*. *pneumoniae* in clinical specimens detected by culture and real-time PCR methods.

	Number of positive results per method		
Number of methods detecting positive results	Culture	Duplex real-time PCR	MpP1 real-time PCR
All three methods	356	356	356
Two methods	-	92	92
	2	-	2
One method	4	-	-
	-	5	-
	-	-	12
Total^a^	362	453^b^	462

^a^ Amounts are total number of positive results out of 1344 clinical specimens

^b^This method detected both *M*. *pneumoniae* types in 3 specimens.

Compared to the aforementioned culture-based typing methods (362/1344), our duplex real-time PCR (453/1344) analysis of the throat swabs identified a greater number of *M*. *pneumoniae* genotypes (paired χ^2^, *P* < 0.01). Among the 453 clinical specimens that tested positive by duplex real-time PCR, both *M*. *pneumoniae* types were identified in 3 specimens (0.7%). MpP1 real-time PCR identified only one of the 3 specimens as positive, and each of these 3 specimens was negative by culture and P1 gene-based typing. Based on genotyping analysis by the duplex real-time PCR assay, 84.4% (385/456 identified genotypes) were type 1, and 15.6% (71/456 identified genotypes) were type 2. A total of 97 culture negative specimens without a P1 gene-based typing result were successfully genotyped with the duplex PCR assay, and only 6 culture-positive specimens genotyped with the P1 gene-based typing method were not detected positive and typed by the duplex real-time PCR assay (Table 5).

From 2008 to 2014, the yearly percentages of *M*. *pneumoniae* type 2 genotypes identified by the P1 gene-based typing method were 11.5% (7/61), 20.0% (4/20), 3.1% (2/64), 9.9% (13/131), 15.2% (5/33), 42.3% (11/26), and 40.7% (11/27), respectively. During the same period, the yearly percentages of *M*. *pneumoniae* type 2 genotypes identified by the duplex real-time PCR assay were 10.8% (8/74), 12.9% (4/31), 5.1% (4/79), 10.9% (18/165), 19.5% (8/41), 45.5% (15/33), and 42.4% (14/33), respectively. The total percentages of *M*. *pneumoniae* type 2 genotypes identified by the P1 gene-based typing and duplex real-time PCR methods during this period were 14.6% (53/362) and 15.6% (71/456), respectively. These genotype percentages were not different from those generated by the culture-based typing methods mentioned above (χ^2^, *P* = 0.7128). Between 2008 and 2012, the percentage of identified *M*. *pneumoniae* type 2 genotypes in clinical specimens from Beijing did not exceed 20%. However, the percentage of type 2 genotypes rapidly increased to over 40% in2013 and 2014 (Table 6).

**Table 6:** *M*. *pneumoniae* genotypes in clinical specimens collected from 2008 to 2014 in Beijing, China.

		Number of *M*. *pneumoniae* genotypes per year						
Typing method	*M*. *pneumoniae* type	2008	2009	2010	2011	2012	2013	2014
P1 gene based method	Type 1	54	16	62	118	28	15	16
	Type 2^a^	7	4	2	13	5	11	11
	Total	61	20	64	131	33	26	27
Duplex real-time PCR	Type 1	66	27	75	147	33	18	19
	Type 2	8	4	4	18	8	15	14
	Total	74	31	79	165^b^	41	33^c^	33
P1 gene and Duplex real-time PCR	Type 1	52	16	61	116	28	15	16
	Type 2^a^	7	4	2	13	4	11	11
	Total	59	20	63	129	32	26	27

^a^Includes type 2 variants.

^b^Both *M*. *pneumoniae* types were detected in two of these specimens.

^c^Both *M*. *pneumoniae* types were detected in one of these specimens.

## Discussion

In this study, we demonstrated that our duplex real-time PCR assay can simultaneously detect and type *M*. *pneumoniae* directly from extracted DNA in clinical specimens, without cultivation of the pathogen. The design of our primers/probes was based on the conserved sequences of the type 1-specific 3kb and type 2-specific 5kb insertion fragments. In the NCBI database, the *M*. *pneumoniae* FH strain (GenBank: CP002077.1) is the only type 2 strain that does not have the 5-kb insertion fragment. Unexpectedly, the resequenced FH strains (GenBank: CP010546.1) had the 5-kb insertion at position *mpna* 5861–5865. Furthermore, the ATCC 15531 (FH) strain that we acquired and used in our analysis still had the 5-kb insertion. This finding was also reported in a previous study by Kenri et al. [36]. Whether the ATCC FH strain in our laboratory and the genome-sequenced FH strain (GenBank: CP002077.1) in the NCBI database are in fact different is unclear.

Results from the paired chi-square analyses of the positive detection rates generated by the culture method and the two real-time PCR methods indicate that the detectability of our duplex PCR assay was similar to that of the MpP1 PCR assay and higher than that of the culture method. Only 6 of the 362 culture-positive throat swabs were detected negative by the duplex real-time PCR assay. After further purification of the *M*. *pneumoniae* DNA from these specimens, we repeated the assay and found that all 6 specimens tested positive and were correctly genotyped. It is likely that the DNA extracts from these clinical specimens initially contained unknown substances that inhibited PCR amplification. Our assessment of the duplex real-time PCR typing ability revealed that the ATCC strain and clinical isolate typing results from the duplex real-time PCR method were in perfect agreement with those from the culture-based methods. Furthermore, duplex real-time PCR analysis (453/1344) of the clinical specimens identified a greater number of *M*. *pneumoniae* genotypes compared to the culture-based typing methods (362/1344), and there were no significant differences in the percentages of each *M*. *pneumoniae* type between the duplex PCR and the culture-based methods. Taken together, these findings demonstrate that this novel duplex real-time PCR assay has high accuracy for genotyping as well as detecting *M*. *pneumoniae* in clinical specimens.

Coinfection with both types of *M*. *pneumoniae* in humans has never been previously reported. In this study, the duplex real-time PCR assay yielded a coinfection rate of 0.7%. However, this rate is likely inaccurate, because analysis of the *M*. *pneumoniae* type 1 and type 2 DNA mixtures revealed that the fluorescent signal corresponding to the DNA type of lower concentration could be quenched by that corresponding to the DNA type of higher concentration. Furthermore, rare strains may exist that contain a single P1 gene, as well as both the 3- and 5-kb insertions. Although our findings do not prove their existence, the possibility of such rare strains cannot be excluded. Unfortunately, *M*. *pneumoniae* strains could not be isolated from 3 specimens. Therefore, the clinical significance of coinfection with both types and the possible existence of a rare strain of *M*. *pneumoniae* remain unclear and require further study.

The annual rates of *M*. *pneumoniae* genotypes in clinical specimens from Beijing revealed that type 1 was the predominate genotype from 2008 to 2012. This trend continued for about 5 years, and then a shift toward type 2 began in 2013 and continued through 2014. In previous years, type 1 was always the predominate genotype in China [37–42]. Therefore to our knowledge, this is the first report of *M*. *pneumoniae* type shifting in China. Of the 53 type 2 strains detected by the culture-based methods, the majority of them were V2c. Unfortunately, the type 2 variants could not detected by duplex real-time PCR. Previous reports have indicated that a certain strain of *M*. *pneumoniae* could confer a type-specific immunity [43]. Although type-specific protection against *M*. *pneumoniae* in response to previous infections in the human population has been reported [1, 44], the relationship between the *M*. *pneumoniae* type shift phenomenon and pneumonia outbreaks needs to be further verified with more extensive surveillance data. This type shift phenomenon has already been reported in Japan and France [12, 15, 45]. Given that, as reported by Kenri et al. [12], a type shift phenomenon occurs every 8–10 years, and a shift from one type to another requires 2–3 years, we can deduce that a shift from *M*. *pneumoniae* type 1 to type 2 in Japan would have occurred around 2012–2013. A recent study by Kubota et al. [46] found that type 1 was still the predominate genotype among *M*. *pneumoniae* strains isolated in Japan between 2011 and 2012. Another report by Ishiguro et al. [47] revealed that the percentage of *M*. *pneumoniae* type 2 isolates in Japan increased from December 1, 2012 to July 31, 2014. To a certain extent, these results support our deduction. Given that Beijing and Japan are located in the same latitude of the East Asia region where there is frequent population migration, it is possible that an *M*. *pneumoniae* type shift phenomenon occurred in these two regions simultaneously. Based on our own findings and inference from the Kenri et al. report, we hypothesize that an *M*. *pneumoniae* type shift phenomenon began in Beijing in 2013 and will last for a few years. Currently, *M*. *pneumoniae* in our population is still in the process of type shifting. Type 2c (V2c) will most likely become the predominate genotype in our population over the next few years. Although *M*. *pneumoniae* type 2 variants have been detected in many regions [38, 47, 48], they comprised a very small percentage of the *M*. *pneumoniae* isolates and never became the predominant genotype. In this study, we found that *M*. *pneumoniae* in our population started shifting from type 1 to 2c at about the same time that this type 2 variant was becoming increasingly prevalent in Japan [47]. Based on these findings, *M*. *pneumoniae* type 2c will likely become the first predominant type 2 variant in the world, perhaps as a result of selection pressure on the human population.

Interestingly, a recent publication failed to find a clear increasing or decreasing trend in P1 type 1, P1 type 2, or their proportion from 2003 to 2012 in Germany [49]. Rather than supporting the hypothesis of type shifts before or during epidemic phases, this result favors the influence of a decreasing level of specific antibodies within the human population as the reason for the start of epidemics. Exactly why these results were different is not clear and requires further study. In any event, we will continue to monitor the status of V2c in Beijing over the next decade. Although the relationship between the type shift phenomenon and pneumonia outbreaks needs to be further verified, increased attention on *M*. *pneumoniae* surveillance in Beijing is warranted.

## Conclusion

Compared to the conventional, culture-based *M*. *pneumoniae* typing methods, our duplex real-time PCR assay has distinct advantages, including time saving and higher typing sensitivity. Although this assay can rapidly differentiate the two *M*. *pneumoniae* types, it is unable to differentiate the variant strains. Based on the findings from this study, this duplex PCR assay is most useful as a rapid surveillance tool for *M*. *pneumoniae* rather than a refined scientific research method. The sensitivity and specificity of the assay should be validated by testing more known *M*. *pneumoniae* isolates in future studies. In conclusion, this novel duplex real-time PCR assay can detect and type *M*. *pneumoniae* in clinical specimens with high sensitivity and accuracy. Our genotyping results provide important information for understanding recent type shifts that can change the epidemiological characteristics of *M*. *pneumoniae* in Beijing.
